# Respiratory outcomes of ultrafine particulate matter (UFPM) as a surrogate measure of near-roadway exposures among bicyclists

**DOI:** 10.1186/s12940-017-0212-x

**Published:** 2017-02-08

**Authors:** Hye-Youn Park, Susan Gilbreath, Edward Barakatt

**Affiliations:** 1 Research Division, California Air Resources Board, 1001 “I” street, P.O. Box 2815, Sacramento, CA 95812 USA; 2 Program in Physical Therapy, College of Health and Human Services, California State University, Sacramento, CA 95819 USA

**Keywords:** Ultrafine particulate matter, Particle number, Bicycle commuters, Lung function

## Abstract

**Background:**

Studies have shown a consistent association between exposure to traffic-related air pollution and adverse health effects. In particular, exposure can be high for cyclists who travel near roadways. The objective of the current study was to examine the relationship between short-term exposure of near-road traffic emissions and acute changes in lung function among individuals who frequently bike in the Sacramento and Davis areas in California. Ultrafine particulate matter (UFPM) was used as a surrogate for near-roadway exposure in this study since the main source of this pollutant is from motor vehicle exhaust.

**Methods:**

Thirty-two bicyclists were recruited and completed two rides on separate days during the study period of March-June, 2008. One ride was on a high traffic route paralleling a section of Interstate 80 (I-80)/Interstate Business 80 (I-80B), and a second one was on a low traffic route, such as bike paths away from major highways. The participant’s lung function was measured before and after each ride, and UFPM exposure was measured during the rides using a condensation particle counter (CPC).

**Results:**

In the final linear mixed-effect model using median UFPM concentrations as the main exposure, we observed that lung function change (post–ride minus baseline measurements) shifted in the negative direction. Lung function changed by 216 mL for FVC and 168 mL for FEV_1_, respectively, for an interquartile range (IQR: 12,225 to 36,833 number of particles/cm^3^) increase of UFPM concentration after adjusting for other covariates of age, sex, wind direction, and day of the week.

**Conclusions:**

This study found significant associations between increased levels of UFPM concentrations as a proxy for near road traffic pollution, and decrements in lung function measurements. Our results are related to short-term exposures, and the long-term health effects of cycling near heavy traffic require further research. Our study suggests the need to reduce traffic pollution, particularly near roads. Cyclists should plan their route to reduce their exposure where possible and further research on built environment designs may help urban planners to reduce the potential health concerns of cyclists’ exposure to traffic-related air pollution.

**Electronic supplementary material:**

The online version of this article (doi:10.1186/s12940-017-0212-x) contains supplementary material, which is available to authorized users.

## Background

Over the past few decades, a large body of scientific evidence has shown a consistent association between exposure to traffic-related air pollution and adverse health effects including the exacerbation of asthma, and increased respiratory symptoms, and cardiovascular diseases [1]. Although traffic pollution is known to be associated with health impacts, the source of these health impacts within the mixture of near-roadway emissions has not been identified. Due to this uncertainty, a few components of traffic air pollution have been utilized as surrogates to investigate the health impacts of exposure including ultrafine particulate matter (UFPM) [2].

Although there are many localized sources of UFPM, the main source of ambient UFPM is combustion from motor vehicles, especially from diesel engines [3–7], and near-roadway exposure dominates total daily exposure to UFPM [8]. While there is no definitive consensus on the best surrogate for traffic pollution, UFPM in the near road environment is dominated by traffic emissions, and has been used as a proxy for exposure to combustion-related traffic emission from vehicles on the roadway [9–11].

Active transportation such as biking or walking has been suggested as an important way of reducing vehicle miles traveled (VMT) and emissions of traffic-related pollutants, as well as providing increases in health benefits from increased physical activity [12, 13]. However, there is a concern that bicycle commuters might experience harmful effects from increased exposures to traffic-related pollution due to their close proximity to vehicle emissions and their increased ventilation rate [14].

Regarding the effects of UFPM from traffic emissions near roadways on cyclists, limited information is available, and the results are not consistent. While Vinzents et al. reported an association between UFPM exposure among cyclists and oxidative DNA damage [11], other studies on subclinical responses such as plasma IL-6, platelet function, or exhaled nitric oxide (NO) did not report any significant changes in cyclist with UFPM exposure [15, 16].

Changes in airway resistance can be seen during pulmonary function testing by measuring the total volume of air an individual can exhale during a maximal breath (FVC) as well as the volume of air that can be exhaled during the first second of a maximal exhalation (FEV_1_). During exercise, changes in the neural input to the lungs allow the smooth muscle that controls airway diameter to relax, effectively reducing the resistance along the airway, and this would be reflected in increases in lung function measurements [17]. On the other hand, breathing polluted air has been shown to activate airway sensory nerves that cause air smooth muscle to contract, which increases airway resistance. This increase in airway resistance can lead to reduction in lung function measurements, which is also seen in many individuals who suffer from obstructive airway diseases such as asthma, chronic obstructive pulmonary disease (COPD), and chronic bronchitis [18].

Zuurbier et al. found that decreased peak expiratory flow (PEF), a measure of large airway function, was observed among healthy nonsmoking adults two hours after UFPM exposure from traffic, but not six hours after exposure, and they did not find any difference in other lung function measurements such as FVC and FEV_1_ [19]. A Canadian study showed a significant association between UFPM exposure and decreased heart rate variability among cyclists with no changes in respiratory function [20]. Also, a Dutch study observed negative trends in lung function measurements such as FVC and FEV_1_ from exposure to traffic air pollutants including UFPM six hours after cycling, and positive associations when lung functions were measured immediately after cycling, but none of these were statistically significant [16]. In addition, one U.S. study conducted in Berkeley, California reported no corresponding changes in lung function after cycling on a high- traffic route compared to a low-traffic route [21]. Similarly, an Australian study didn’t find significant differences in lung function or other related airway inflammation measures between higher and lower proximity to motorized traffic, and they observed a significant decrease in UFPM with decreased proximity to traffic compared to increased proximity [22].

Given the limited number and inconsistent findings of previous studies in California, we examined the relationship between short-term exposure to UFPM, as a surrogate for short-term exposure to near-road traffic emissions, and acute changes in lung function among routine bicyclists in the Sacramento/Davis region of California.

## Methods

### Study design

We recruited 32 bicyclists in the Sacramento area through local bicycle organizations, office bicycle commuter groups, and word-of-mouth. These subjects routinely used cycling for active transportation and most rode bicycles to work or to run errands on a regular basis, at least three days a week. Subjects with preexisting conditions such as eye, thoracic or abdominal surgery, a myocardial infarction, unstable angina pectoris, or a history of syncope associated with forced exhalation, were excluded unless they obtained a medical release from their physician.

We asked participants to complete two rides on separate days during the study period of March, 2008 through June, 2008. One ride was a high traffic route, directed by researchers, paralleling a section of Interstate 80 (I-80) /Interstate Business 80 (I-80B) from the point where this highway crosses Mace Boulevard in Davis to the area where the highway crosses 3^rd^ street in Sacramento, California. For approximately 12 km of the high traffic route the cyclists were within 10–20 m of I-80 (Rural Interstate), and for another 10 km of the route the cyclists were within 400 m of the I-80/I-80B corridor (Urban Interstate) on heavily used local thoroughfares (Major Collectors).

The other routes were low traffic routes that included minor local roads (Urban Minor Collectors), Rural Local Roads, and/or bike paths away from major highways. On the low traffic route participants rode at a speed similar to that ridden on the high traffic route. High traffic routes had over 100,000 vehicles per day on average (annual average daily traffic) [23].

The high traffic route, assigned by researchers, was mostly uniform across subjects and took place from home to workplace which included a ride across the causeway and on thorough-fare (Major Collectors) parallel to urban and rural interstate sections. However, the low traffic routes were more varied and were selected for convenience, in consultation with the researchers. All participants provided written informed consent, and the study was approved by the State of California Institutional Review Board, the Committee for the Protection of Human Subjects.

### Exposure measurements

To measure exposure to ultrafine particulate matter concentrations, a condensation particle counter (CPC) (TSI Model 3007, TSI, St. Paul, MN, USA) was carried in a handle bar rack on the researcher’s bike. The particle counter used a continuous measurement (an interval of 5 s) of the particle number concentration of ultrafine particles sized down to 10 nm. Because particle number concentration is dominated by the smallest particles, this can be considered a measure of UFPM. The CPC instrument was calibrated by the manufacturer and a researcher performed flow checks before conducting the study. The researcher accompanied the study participants who selected the pacing on each ride. After each ride, the stored data were downloaded onto the study computer.

Ambient air pollution (PM2.5, NO_X_, NO_2_, NO, and O_3_) and meteorological data were obtained from National Air Monitoring Stations/State and Local Air Monitoring Stations (NAMS/SLAMS) in the Sacramento and Davis areas.

### Respiratory outcome measurements

Immediately before and after each ride the lung function of the participants was measured using a portable spirometer (EasyOne Frontline: Medical Technologies Andover, MA, USA). The results were stored in the spirometer and downloaded onto the password-protected computer. Forced vital capacity (FVC), forced expiratory volume in one second (FEV_1_), FEV_1_/FVC, and peak expiratory flow rate (PEF) were used as indicators of lung function outcomes. Also, the pulse rate of the participants was recorded before and after each ride using a pulse oximeter (Onyx 9500 Finger Pulse Oximeter, NONIN Medical, Inc., Plymouth, MN, USA).

### Statistical analyses

The difference in lung function between pre- and post-ride for each subject was calculated as an outcome variable. Median concentrations of UFPM exposure (number of particles/cm^3^) was used for the main exposure variable after natural log transformation, and for the purpose of interpretation of the model results, the IQR of UFPM exposure concentrations was used to calculate the change in magnitude for lung function outcomes.

For the first part of the statistical analyses, we compared the UFPM median concentration by route type (high traffic vs. low traffic route). The lung function differences between a high traffic route and a low traffic route by subject were also compared. If the data were normally distributed, we used a paired *t*-test, otherwise, the non-parametric Wilcoxon Rank-Sum test was used. Additionally, we analyzed the associations between the median concentration of UFPM exposure during cycling and the difference in lung function (post-ride level minus baseline ride level) using mixed models with random effects of subjects to account for correlation between measurements from the same participant.

Other covariates including age, sex, body mass index (BMI), race, average ambient temperature, average humidity during cycling, wind direction (downwind vs. non-downwind), wind speed, pulse rate difference, average cycling speed, distance of ride, time of the day for cycling (morning vs. afternoon), and day of the week (weekdays vs. weekend) were evaluated. In a multivariate analysis using linear mixed-effect models, we developed a statistical model with a broad inclusion of all covariates, and used a 10% change-in-estimate (change > 10%) as the criterion for deciding which confounding factors to exclude [24]. Covariates selected for the final models were sex, age, wind direction, and the day of the week.

The SAS statistical package was used for analysis (Version 9.3, SAS Institute, Cary, NC).

## Results

A total of 32 frequent bicyclists participated in the study (Table 1). The majority of study subjects were male (75%, *n* = 24) and non-Hispanic white (91%, *n* = 29), with a small percentage of Asian (6%, *n* = 2), and one participant that didn’t specify the ethnic information in the questionnaire. The mean age of the participants was 45.1 years (SD: 12.5 years), and a healthy body weight was reported for the participants based on BMI (mean: 24.5, SD: 2.4). On average, participants rode 22.2 km (13.9 miles) for each trip in this study. Within the group of study subjects, 4 (13%) subjects reported being former smokers. Four participants (13%) reported a history of asthma, and 17 (53%) responded “yes” to a history of allergies.

**Table 1:** Descriptive characteristics of the participants (*n* = 32)

Characteristics	Number (%), SD^a^
Sex- n (%)	
Male	24 (75%)
Female	8 (25%)
Race/ethnicity - n (%)	
White	29 (91%)
Asian	2 (6%)
Not specified	1 (3%)
Age- Mean (range)	45.1 (23–68), SD^a^: 12.5
Height (cm) -Mean (range)	175.0 (155.0–196.0), SD: 9.0
Weight (kg) -Mean (range)	75.3 (51.3–108.9), SD: 11.7
BMI^b^- Mean (range)	24.5 (20.1–29.8), SD: 2.4
Distance for cycling (km)- Mean (range)	22.2 (16.0–32.0), SD: 3.1
Former smokers - n (%)	4.0 (13%)
Ever diagnosed as asthma- n (%)	4.0 (13%)
Ever diagnosed for allergies- n (%)	17.0 (53%)

^a^ *SD* Standard deviation

^b^ *BMI* Body weight (kilograms) divided by the square of the body height (meters)

Table 2 shows the distribution of UFPM concentrations in number of particles per cubic centimeter (number of particles/cm^3^) route type. We observed that the mean concentrations were nearly three times higher on the high traffic routes compared to those on the low traffic routes (49,369 vs. 17,474 in number of particles/cm^3^). This difference in average concentration of UFPM between road types was statistically significant (*p* < 0.0001) with Wilcoxon Rank -Sum test.

**Table 2:** Distribution of UFPM particle number concentration (in number of particles/cm^3^) by route type (*n* = 32)

Road type	Mean ± SD	Median	Range (Min-Max)
High traffic routes	49,369 ± 11,812	44,023	36,049–98,420
Low traffic routes	17,474 ± 6,600	16,553	7,694–25,924

We measured lung function before cycling (baseline) and right after cycling (post) for each ride (Table 3). For FVC, the average change (post minus baseline) in lung function for low traffic routes and for high traffic routes was +0.14 l (SD: 0.31) and −0.12 l (SD: 0.33), respectively, and these were statistically significant (*p* = 0.004). Similarly, we found a significant increase of 0.11 l (SD: 0.18) in FEV_1_ after rides for low traffic routes (*p* = 0.002) while a 0.06 l (SD: 0.19) non-significant decrease in FEV_1_ was observed for high traffic routes. The differences in the effects of high traffic and low traffic routes on lung function for FVC and for FEV_1_ were statistically significant (*p* = 0.0005 and *p* = 0.003) respectively from either a Wilcoxon Rank-Sum test or a paired *t*-test. There were no statistically significant differences in lung function changes between route types for FEV_1_/FVC ratio or PEF.

**Table 3:** ^a^Mean and difference of lung function measurements by time of measure and by route type (*n* = 32)

Outcomes	Low traffic routes			High traffic routes		
	Pre-ride^a^	Post-ride	Diff^b^	Pre-ride	Post-ride	Diff
FVC (liters)**	4.74 (0.91)	4.89 (0.96)	0.14 (0.31)	4.86 (0.90)	4.73 (0.99)	−0.12 (0.33)
FEV_1_ (liters)**	3.80 (0.79)	3.91 (0.79)	0.11 (0.18)	3.86 (0.78)	3.81 (0.80)	−0.06 (0.19)
FEV_1_/FVC	0.80 (0.05)	0.80 (0.05)	0.00 (0.04)	0.80 (0.05)	0.81 (0.06)	0.01 (0.04)
PEF (liters/min)	588.57 (119.93)	575.54 (130.93)	−13.03 (52.02)	584.83 (123.45)	568.38 (126.71)	−16.45 (49.98)

**< 0.005 (significant difference in average lung function measure between low traffic routes and high traffic routes

^a^Mean (SD)

^b^Diff = Post-ride minus Pre-ride (SD)

Table 4 shows the parameter estimates of median UFPM concentrations related to lung function measures from the final linear mixed-effect model adjusted for the covariates of age, sex, wind direction, and day of the week. We observed significant associations between increased levels of median UFPM concentration and decreased lung function in FVC and FEV_1_ (*p* < 0.01), but not with FEV_1_/FVC ratio or PEF. The interpretation of these significant estimates is that lung function change (post minus baseline) shifted in the negative direction by 216 mL (3.9%) for FVC and 168 mL (4.1%) for FEV_1_, respectively, for the IQR increase of median UFPM concentration (12,225 to 36,833 number of particles/cm^3^), based on parameter estimates shown in Table 4 (*β* = −0.196, and *β* = −0.153, respectively). Regarding other covariates (data shown in the Additional file 1: Tables S1 and S2), weekdays compared to weekends as a reference showed significant associations (*β* = −0.343 for FVC, and *β* = −0.175 for FEV_1_) with decreased lung functions. Thus, weekdays compared to weekends might reflect collectively the effects of other unmeasured pollutants or other factors on lung functions. Also, we observed a negative trend of downwind direction in comparison with non-downwind for lung function changes, however, it was not statistically significant (*β* = −0.065 for FVC, and *β* = −0.016 for FEV_1_). In the final full regression model, sex, age, wind direction (downwind vs. non-downwind), and the day of the week (weekdays vs. weekends) were adjusted as confounders.

**Table 4:** Regression coefficients^†^ of associations between median concentrations of UFPM exposure and lung function changes (post-pre)

Outcomes	Estimate	95% Confidence Interval
FVC (liters)	−0.196**	(−0.314, −0.077)
FEV_1_ (liters)	−0.153***	(−0.221, −0.084)
FEV_1_/FVC	0.002	(−0.009, 0.013)
PEF (liters/min)	−3.103	(−15.390, 9.183)

Regression coefficients of natural log-transformed median concentration of UFPM exposure after adjusting for age, sex, wind direction (downwind vs. non-downwind), and day of the week (weekdays vs. weekend) variables

** *p* < 0.005; *** *p* < 0.0001

## Discussion

We found a significant association between decreased lung function and short-term exposure to UFPM measured during cycling. Previously, Strak et al., reported positive trends in associations with lung function changes immediately after cycling, but negative associations six hours after cycling though none of these results reached statistical significance [16]. Another study showed that a decrease in PEF with relation to UFPM exposure was observed right after cycling, however, similar results were not found with other lung function measurements such as FVC and FEV_1_ [19].

In this study, nearly three times higher UFPM concentrations were observed in high traffic routes compared to low traffic routes. High traffic routes (e.g., I-80 and I-80B) have high traffic flows, over 100,000 vehicles per day on average [23], thus, proximity to a high traffic volume of motor vehicles is reflected by the observed increase in UFPM concentrations. When compared to previously published studies, UFPM concentrations observed in the current study were almost double, and the bike routes used by cyclists were 2 to 4 times longer [15, 16, 20, 21]. Thus, the lower level of UFPM exposure seen in more recently conducted studies and shorter bike routes might be a reasonable explanation for why these studies with cyclists did not observe similar significant reductions in pulmonary function to those seen in our study. In our study, we observed that lung function change (post minus baseline) shifted in the negative direction by 216 mL (3.9%) in FVC and 168 mL (4.1%) in FEV_1_ for IQR increase of median UFPM concentration, and the magnitude of these changes in mL may represent a small increase in airway resistance.

In addition, the participants in our study used cycling frequently for active transportation and some of their typical daily commuting routes included areas along the major highways and roads in the Sacramento and Davis regions. Since some of these routes were used in this study, the study design reflected a real-life situation rather than scripted exposure designs, used more commonly in the previously published studies.

There are limitations for this study. First, other air pollutants during the bicycle rides were not concurrently measured using a personal exposure monitor. As a crude proxy for personal exposure of other pollutants, we tested two pollutant models using ambient air pollution data from nearby monitoring stations (PM2.5, NO_X_, NO_2_, NO, and O_3_). The additional co-pollutant modeling approach did not change the estimates of UFPM concentration on lung function, and these other pollutants didn’t show significant associations. However, considering the fact that these other pollutants were measured only at monitoring stations not during bike rides, we cannot make an inference of these pollutants’ impacts on lung functions per se. Since our findings do not specifically show a relationship solely with UFPM exposure, it is still possible that other pollutants, especially other near roadway traffic and toxic pollutants as well as other unmeasured factors associated with traffic, may have contributed to the results of decreases in lung function collectively. Although some evidence has suggested that adverse respiratory effects may be attributable to smaller particles such as UFPM [2, 25, 26], the source of these health effects from the mixture of near-roadway emissions remains unidentified.

Asthmatics and smokers can have increased impacts from traffic exposure; both cases of asthmatics and former smokers were included in our study. As part of a sensitivity analysis, we repeated the statistical analyses after excluding asthmatic cases (*n* = 4) or former smokers (*n* = 4), and similar results were obtained (e.g., *β* = −0.186 for FVC, and *β* = −0.159 for FEV_1_, respectively). Also, we included allergy histories in the final model development, and it was not statistically significant.

Additionally, airway inflammation is a good biomarker for respiratory diseases, and certain indicators such as exhaled nitric oxide (eNO) for airway inflammation can be informative to confirm lung function symptoms sub-clinically [27]. Some previous research with healthy cyclists showed mixed findings (either negative or positive associations with eNO) after exposed to air pollutants such as UFPM during cycling [15, 16], therefore, more research on airway inflammation along with pulmonary function is warranted.

Apart from air pollution, traffic produces noise which can influence health as a stressor. Previous studies have shown that long-term exposure to traffic noise is associated with an increased risk of diabetes [28] or cardiovascular disease [29]. The current study evaluated the effects of short-term exposure to UFPM, unlike most of the noise-related health research which focused on long-term exposure effects. Hence, the role of noise as a possible confounder in this study is unclear at this time.

## Conclusions

Our study showed significant associations between increased levels of UFPM concentrations, measured as a surrogate for short-term exposure of near road traffic emissions, and decreased lung function after adjusting for the covariates of age, sex, wind direction, and day of the week. Previously, the beneficial effects of physical activities such as cycling have been shown to outweigh the risk of air pollution exposure effects or traffic accidents in modeled analyses [30]. Although further research will be necessary to evaluate the impacts of chronic traffic-related UFPM exposure on cyclists, it is reasonable to assume that adverse impacts from the cumulative effects of repeated short-term exposures to traffic pollution may be significant in those that cycle frequently for longer periods of time. There is also a need for more research to investigate the constituents of traffic pollution (e.g., UFPM, elemental carbon, brake wear, tire wear, noise) to determine the factors contributing to its adverse health effects.

Additionally, the majority of the study subjects were healthy white males, therefore, the generalizability of the findings to other populations must be interpreted with caution. Also, larger adverse impacts on lung function may be more likely in more sensitive populations such as older individuals or those with more pronounced respiratory conditions.

Overall, our study suggests the need for urban planners to consider the public health concerns of cyclists and their exposure to traffic-related air pollution. Reducing traffic emissions in the environment is critical, particularly on and near roads where cyclist travel. Research may help to determine effective mitigation strategies through built environment designs that reduce cyclist exposure to traffic pollution and promote safe active transportation. Also, cyclists should plan their route to reduce their exposure to traffic pollution by cycling away from heavily trafficked roads and highways, where possible.

## Additional file

Additional file 1: The file contains 2 supplemental tables with full model specifications referenced in the main manuscript text. **Table S1.** Final full multiple regression model with FVC (post-pre). **Table S2.** Final full multiple regresion model with FEV (post-pre). (DOCX 16 kb)

## Abbreviations

CPC: Condensation particle counters
FEV_1_: Forced expiratory volume in one second
FVC: Forced vital capacity
IQR: Interquartile range
PEF: Peak expiratory flow rate
UFPM: Ultrafine particulate matter
