# Supplementary material for: Respiratory outcomes of ultrafine particulate matter (UFPM) as a surrogate measure of near-roadway exposures among bicyclists
Source: Environ Health. 2017 Feb 8;16:6. doi: 10.1186/s12940-017-0212-x (PMC5299642; doi:10.1186/s12940-017-0212-x)
Supplement: Additional file 1: — The file contains 2 supplemental tables with full model specifications referenced in the main manuscript text. Table S1. Final full multiple regression model with FVC (post-pre). Table S2. Final full multiple regresion model with FEV (post-pre). (DOCX 16 kb) [file 12940_2017_212_MOESM1_ESM.docx]

**S.1. Final full multiple regression model with FVC (post-pre).**

| **Variables** | **Estimate (95% CI)** | |  |
| --- | --- | --- | --- |
| **UFPM** (in natural log-transformed) | -0.196 (-0.314, -0.077)** | |  |
| **Sex :** Female vs. Male(reference) | -0.413 (-0.633, -0.193) ** | |  |
| **Age** | -0.001 (-0.009, 0.006) | |  |
| **Wind direction:** downwind vs. non-downwind (reference) | -0.065 (-0.243, 0.113) | |  |
| **Day of the week :** weekdays vs. weekend (reference) |  | 0.--0.343 (-0.535, -0.151) ** | |
|  |  |  | |

^†^ Regression coefficients of natural log-transformed median concentration of UFPM exposure after adjusting for age, sex, wind direction (downwind vs. non-downwind), and day of the week (weekdays vs. weekend) variables.

***p*< 0.005

**S.2. Final full multiple regression model with FEV_1_ (post-pre).**

| **Variables** | **Estimate (95% CI)** | |  |
| --- | --- | --- | --- |
| **UFPM** (in natural log-transformed) | -0.153 (-0.221, -0.084)*** | |  |
| **Sex :** Female vs. Male(reference) | -0.148 (-0.275, -0.021)* | |  |
| **Age** | -0.001 (-0.003, 0.005) | |  |
| **Wind direction:** downwind vs. non-downwind (reference) | -0.016 (-0.119, 0.087) | |  |
| **Day of the week :** weekdays vs. weekend (reference) |  | 0.--0.175 (-0.286, -0.065) ** | |
|  |  |  | |

^†^ Regression coefficients of natural log-transformed median concentration of UFPM exposure after adjusting for age, sex, wind direction (downwind vs. non-downwind), and day of the week (weekdays vs. weekend) variables.

**p*< 0.05; ** *p*< 0.005; *** *p*< 0.0001
